# Interplay between Cellular and Molecular Inflammatory Mediators in Lung Cancer

**DOI:** 10.1155/2016/3494608

**Published:** 2016-01-28

**Authors:** Mario Orozco-Morales, Giovanny Soca-Chafre, Pedro Barrios-Bernal, Norma Hernández-Pedro, Oscar Arrieta

**Affiliations:** ^1^Experimental Oncology Laboratory, National Cancer Institute of Mexico (INCan), 14080 Mexico, DF, Mexico; ^2^Thoracic Oncology Unit, National Cancer Institute of Mexico (INCan), 14080 Mexico, DF, Mexico

## Abstract

Inflammation is a component of the tumor microenvironment and represents the 7th hallmark of cancer. Chronic inflammation plays a critical role in tumorigenesis. Tumor infiltrating inflammatory cells mediate processes associated with progression, immune suppression, promotion of neoangiogenesis and lymphangiogenesis, remodeling of extracellular matrix, invasion and metastasis, and, lastly, the inhibition of vaccine-induced antitumor T cell response. Accumulating evidence indicates a critical role of myeloid cells in the pathophysiology of human cancers. In contrast to the well-characterized tumor-associated macrophages (TAMs), the significance of granulocytes in cancer has only recently begun to emerge with the characterization of tumor-associated neutrophils (TANs). Recent studies show the importance of CD47 in the interaction with macrophages inhibiting phagocytosis and promoting the migration of neutrophils, increasing inflammation which can lead to recurrence and progression in lung cancer. Currently, therapies are targeted towards blocking CD47 and enhancing macrophage-mediated phagocytosis. However, antibody-based therapies may have adverse effects that limit its use.

## 1. Non-Small Cell Lung Cancer (NSCLC)

Lung cancer remains the leading type of cancer worldwide and in Latin America [1, 2]. The disease burden is significantly high, with around 2.5 million new cases per year and 1.5 million deaths worldwide [3]. The two main histological subtypes of lung cancer are small-cell lung cancer (SCLC), which comprises 15% of cases, and non-small-cell lung cancer (NSCLC) accounting for 85% of cases [4] which include adenocarcinoma, squamous cell carcinoma, and large cell carcinoma [5]. Among all newly diagnosed NSCLC cases, adenocarcinomas are the most frequent subgroup following by squamous cell carcinomas [6, 7]. Cigarette smoking is the major risk factor for lung cancer but around 10–20% of cases are found in never smokers; also wood-smoke is a major risk factor in countries like Mexico [8–11].

Surgery is the selected treatment for early stage NSCLC with the greatest probability of long-term survival in such patients [12]. In advanced NSCLC, conventional therapies are based on chemotherapy and radiotherapy but with low efficacy. Over the last decade, there have been advances in the study of molecular pathways underlying tumor development leading to the development of targeted therapies such as tyrosine kinase inhibitors (TKIs) and antibodies directed against the two main actionable genes in NSCLC up to now: mutations in the epidermal growth factor receptor (EGFR) gene targeted by TKIs like gefitinib [13, 14], erlotinib [9, 15, 16], and afatinib [17–19] and translocations involving the anaplastic lymphoma kinase (ALK) gene treated with the TKI crizotinib [20], alectinib [21], and ceritinib [22]. Benefits have been shown in a subset of 15–20% of patients harboring EGFR mutations which correlate with definite clinical characteristics: adenocarcinoma histology, female sex, Asian ethnicity, and nonsmokers [23–25]. Despite these improvements in therapeutic strategies, early diagnosis is very difficult; most cases are diagnosed at an advanced stage and cancer metastasis is very frequent; therefore, there is still an exceedingly low 5-year survival rate of 11–24% [26–28].

The immunotherapy approach has opened new therapeutic options in advanced NSCLC with the advent of antibodies against immune checkpoints [29, 30]. Recently, the anti-programmed death-1 (PD-1) antibodies nivolumab and pembrolizumab have been approved in the treatment of advanced metastatic NSCLC based on results from clinical trials after prior chemotherapy [31, 32]. Both antibodies block signaling through PD-1 and may restore antitumor immunity with benefits in overall survival [33, 34]. For example, nivolumab, a fully human monoclonal antibody, has recently shown greater overall survival than docetaxel [35]. Pembrolizumab has demonstrated safety and efficacy as single agent for the treatment of NSCLC [32]. These antibodies exhibit a reasonable toxicity profile but they should be administered in selected patient populations based on biomarkers such as PD-L1 expression to avoid serious immune-mediated adverse effects [36]. Although these checkpoint inhibitors have proven efficacy in patients, their mechanism of action implies side effects as the onset of autoimmune diseases and a series of endocrine disorders [37, 38]. This is the rationale for further research into other molecular and cellular factors of the immune system that could be effectively targeted to develop novel therapeutic strategies for the management of advanced NSCLC.

Recent findings indicate that inflammation plays a key role in tumor progression and survival across several cancer types [39]. Cancer related inflammation affects many aspects of malignancy including proliferation, survival, angiogenesis, and tumor metastasis [40]. Inflammatory components in the development of the neoplasm include diverse leukocytes populations, like macrophages and neutrophils, which respond immediately to inflammatory stimulus [41]. Immunoregulatory cytokines secreted in a proinflammatory environment also contribute to tumor growth and metastases and identify patient subsets in advanced NSCLC with differential prognosis [42]. Both macrophages and neutrophils are increased in patients with lung cancer; this is associated with poor clinical outcomes, suggesting that these cells might have important tumor-promoting activities [43, 44]. Tumors escape phagocytosis and immune response through overexpressing CD47 that interacts with the signal regulatory protein alpha (SIRP*α*) preventing engulfment [45]. Their effects are mediated through complex regulatory networks. Human cytokine profiles could define patient subgroups and represent new potential biomarkers.

## 2. Tumor-Associated Macrophages (TAMs)

Macrophages within the tumor microenvironment are called tumor-associated macrophages (TAMs). TAMs have a complex relationship with tumor cells; at an early stage they attack tumor cells avoiding tumor spread; however, over time they begin producing reciprocal growth factors and establish a symbiotic relationship with tumor cells [46]. Macrophages are polarized into two functionally distinct forms M1 and M2, mirroring the Th1 and Th2 nomenclature of T cells [47]. Differentiation of the M1 macrophages is induced by interferon-*γ*, lipopolysaccharides, tumor necrosis factor (TNF) *α*, and granulocyte-monocyte colony-stimulating factor. The M1 macrophages produce high levels of interleukin- (IL-) 12, IL-23, TNF*α*, IL-1, IL-6, CXC ligand 10 (CXCL10), inducible nitric oxide synthase (iNOS), human leukocyte antigen- (HLA-) DR, and reactive oxygen and nitrogen intermediates [47, 48]. Differentiation of the M2 macrophages is induced by IL-4, IL-10, IL-13, IL-21, activin A, immune complexes, and glucocorticoid [47]. The M2 macrophages express high levels of IL-10, IL-1 receptor antagonist, CC ligand 22 (CCL22), scavenger, mannose receptor, galactose receptor, arginase I, and CD163 antigen, reduce the expression of iNOS, and inhibit antigen presentation and T cell proliferation [47, 49].

Factors that shift TAMs towards a M2 phenotype include the location of TAMs within the tumor microenvironment, tumor stage, and type of cancer. Nevertheless, it is still not fully defined whether the diversity within the TAM population is due to the maturation of unique monocytic precursors or due to various factors within the local tumor microenvironment [50]. The M2 macrophages have been found to encourage the growth of various tumour cells* in vitro* and to increase tumor cell survival [51, 52]. M1 macrophage significantly decreased A549 cell viability and proliferation as well as invasion ability [53].

Studies suggest that in solid tumors established and progressively growing TAMs are reprogrammed to induce immune suppression* in situ* in the host through cytokines, prostanoids, and other humoral mediators [54, 55]. Tumor microenvironment can influence the functional status of macrophages* in situ* [56]. IL-1 and IL-6 expression in TAMs differs in ovarian cancer compared to peripheral blood monocytes. TAMs in the ovary produce low levels of IL1 and increase the release of IL-6, which contributes to elevated acute phase proteins and increased malignancy [55].

There is an association between the number of macrophages and prognosis in a variety of human tumors. TAM infiltration increased in carcinomas of breast, cervix, and bladder and correlates with a poor prognosis. However, in prostate, lung, and brain, increasing TAMs is associated with regression of tumors [46].

TAMs can regulate the development of new blood vessels within tumors. In hypoxic sites, they stimulate the production of enzymes and extracellular matrix molecules that regulate endothelial cell activity by stimulating factors such as vascular endothelial growth factor (VEGF), basic fibroblast growth factor (bFGF), tumor necrosis factor-*α* (TNF-*α*), transforming growth factors- *α* and *β* (TGF- *α*, *β*), interferons, thrombospondin, IL-8, and epidermal growth factor (EGF) [57].

## 3. Tumor-Macrophage Interactions in Lung

Innate immunity in lung involves alveolar macrophages (AMs) which act as a barrier avoiding penetration of pathogens. Conversely, macrophages contribute in part to the pathogenesis of lung disease due to toxic particles ingestion, releasing lysosomal enzymes that can kill the macrophage itself, or contribute to the recruitment of new macrophages inducing chronic inflammation [58]. Clinical evidence has indicated that the activation of alveolar macrophages by SiO2 produces rapid and sustained inflammation characterized by the generation of monocyte chemotactic protein 1, which, in turn, induces fibrosis [59].

**Figure 1:**
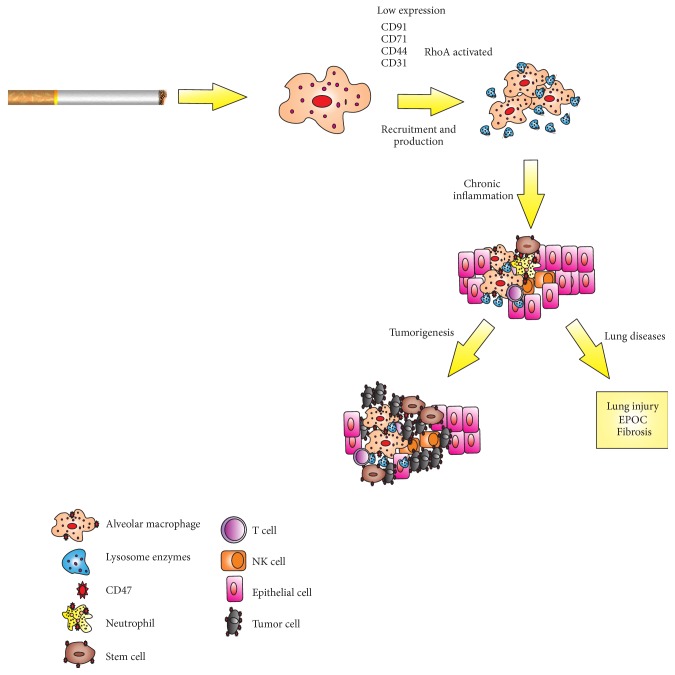
Smoke exposure mediated pathogenesis of pulmonary disease. The exposition to cigarette smoke by macrophages leads to release of lysosomal enzymes able to inhibit phagocytosis by macrophages. Activation of alveolar macrophages deregulates expression of adhesive molecules (CD36, CD91, and CD44) and activated RhoA inhibiting efferocytosis. The rapid and sustained inflammation may contribute to the lung injury and tumorigenesis.

Exposure to cigarette smoke activates NF-E2-related factor 2 (Nrf2) in macrophages and reduces neutrophil recruitment, reduces AMs phagocytic ability and expression of several important recognition molecules, and impairs clearance of apoptotic cells through oxidant-dependent activation of RhoA [60, 61]. In current smokers, the exposure to cigarette smoke affects several important recognition molecules on AMs and downregulates CD31, CD91, CD44, and CD71 on these cells [60]. AMs with defective phagocytosis lead to chronic inflammation and significantly increase the likelihood of developing chronic obstructive pulmonary disease, lung injury, and cancer [62] (Figure 1).

**Figure 2:**
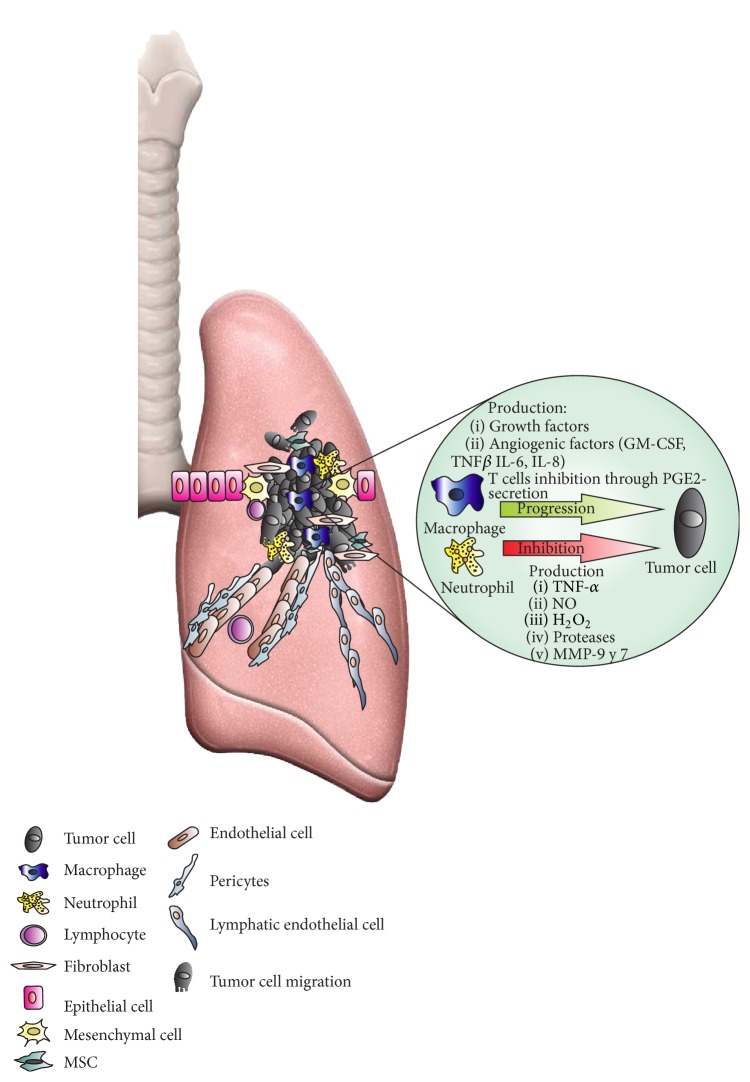
Inflammation: a component of the tumor microenvironment. During malignant transformation until progression disease, the recruitment of immune cells and secretion of soluble factors play an important role in tumor genesis. Tumor killing is promoted for proinflammatory microenvironment where polarized M1 macrophages and N1 neutrophils are recruited. The production of soluble factors, such as TNF-*α*, NO, H_2_O_2_, proteases, and metalloproteinase by immune cells, inhibits tumor growth. However, the generation of an anti-inflammatory environment and the alternative activation of M2 macrophages and N2 neutrophils promote tumor growth. Also, growth factors and angiogenic factors (GM-CSF, TNF-*β*, IL6, and IL8) contribute to tumor proliferation and the inhibition of immune response through prostaglandins.

The infiltration of alveolar macrophages promotes the death of tumor cells in those sites of primary tumor growth and/or metastasis in lung [63]. The antitumor activity of alveolar macrophages from lung cancer patients decreases with increased metastasis, tumor size, and development of pleural invasion [64]. The onset of malignant disease triggers the immune response recruiting TAMs into the tumor site. High numbers of intratumor TAMs have been linked with invasion, angiogenesis, hypoxia, and early occurrence of metastasis in different tumor types including lung cancer [48, 50] (Figure 2).

In patients with NSCLC, the M1 macrophage phenotype has been associated with the expression of IL-1, IL-12, tumor necrosis factor-*α* (TNF-*α*), and iNOS and also has been correlated with extended survival time [65]. In a study, M1 TAMs were identified using CD68 and iNOS markers in tumor compared to nontumor tissue in NSCLC patients. Results indicate that iNOS expression is lower in tissues from patients with adenocarcinoma and squamous cell carcinoma compared to nontumor tissues but surprisingly this was not the case in large cell lung carcinomas [66]. The classically activated M1 macrophages produce effector molecules such as reactive oxygen intermediates, reactive nitrogen intermediates, and TNF*α*, to limit tumor growth. Overall there is an association of M1 TAMs with better lung cancer prognosis.

At the other end are the alternatively activated M2 macrophages which have been correlated with tumor initiation, progression, metastases, by secretion of matrix-degrading enzymes, angiogenic factors, and immunosuppressive cytokines chemokines, inhibiting inflammation [65, 67, 68]. M2 macrophages polarized by cigarette smoke lead to proliferation, migration, and invasion of alveolar basal epithelial cells, and exposition to these cigarette smoke-induced M2 macrophages also significantly increased the cell population in G2/M phase causing proliferation in lung cancer cells [69].

Patients with combination gene signature of M1/M2 macrophages exhibited high median overall survival [53]. In NSCLC, the concentration of macrophages M2 was 70% in comparison with 30% M1. Density of macrophages M1 in the tumor islets, stroma, or islets and stroma was positively associated with patient's survival time [66]. Also, M1 in islet is a predictive response value to survival [66].

## 4. Tumor-Associated Neutrophils (TANs)

Neutrophils are also polarized into N1/N2 subgroups, N1 being proinflammatory, while N2 is anti-inflammatory. N1 and N2 represent a dichotomy in neutrophil subpopulations present in patients and animal models with cancer where they play distinctive roles in the pathogenesis of disease [70]. TANs have a complex interaction with T cells in the tumor microenvironment [71]. They displayed an activated phenotype that included chemokine receptors as CCR5, CCR7, CXCR3, and CXCR4. Also, TANs produced proinflammatory factors MCP-1, IL-8, MIP-1*α*, and IL-6, as well as the anti-inflammatory IL-1R antagonist [72]. Also, TANs exhibit high activated phenotype compared with peripheral neutrophils. In cancer patients, TANs could drive antitumoral immunity through regulating cytotoxic T lymphocytes. In early stages of lung cancer disease, TANs increased T cell IFN-*γ* production and activation and increase T cell proliferation [72]. The blockage of TGF-*β* is able to polarize N2 TANs to N1 TANs in murine models of mesothelioma and lung cancer [73].

Resolution of inflammation involves cessation of neutrophils recruitment and initiation of apoptosis and clearance [74]. If apoptotic neutrophils within the tissues are not removed in an efficient and timely manner, they will become necrotic and release cytotoxic granule proteins that may perpetuate host tissue damage. Thus, neutrophils apoptosis and clearance is a critical limiting factor for the successful resolution of inflammation [75]. In colon adenocarcinoma cell line, massive infiltration of neutrophils showed regression of tumor [76].

So far, the possible mechanisms by which neutrophils are increased in NSCLC patients have not been described; despite this, these cells are dysfunctional [77]; increased levels of IL-8 could explain this accumulation; however, the mechanisms by which this occurs are not known [42].

## 5. CD47 and Immune Evasion

Chronic inflammation confers higher risk of developing cancer. Neutrophils are recruited to tumor sites through transendothelial migration involving the CD47:SIRP*α* recognition (signal regulatory protein alpha) creating an inflammatory environment [78]. Malignant cells escape phagocytosis displaying high levels of CD47 on their surface which binds to SIRP*α* in macrophages and dendritic cells. After binding to SIRP*α*, CD47 induces a dephosphorylation cascade preventing phagocytosis through impaired synaptic myosin accumulation [79]. In this way, CD47 can regulate the function of cells in the monocyte/macrophage lineage [80–82].

CD47 is a ubiquitous cell-surface molecule from the immunoglobulin (Ig) superfamily that interacts with SIRP*α*, thrombospondins, and integrins [83]. CD47 was first isolated in association with integrin in neutrophil granulocytes and was later shown to regulate integrin function [84, 85]. It plays a role in cellular processes like proliferation, apoptosis, adhesion, and migration [86] and in immunological processes such as inflammatory response, immune response, and tumor immunity [87, 88]. This receptor is recognized as a marker of “self” [89] highly expressed by circulating hematopoietic stem cells, red blood cells, macrophages, macrophages neutrophils, and many cancer types [90]. CD47 has also been identified as a tumor marker, and its dysregulation contributes to cancer progression and evasion of antitumor immunity [91–94].

CD47 is expressed ubiquitously whereas its counter-receptor SIRP*α* is more abundant in myeloid-lineage cells such as macrophages, neutrophils, and dendritic cells [95]. Several processes are regulated through the CD47:SIRP*α* signaling system of macrophages, including phagocytosis mature red blood cells (RBCs) in the spleen, phagocytosis of senescent cells and apoptotic bodies, rejection of transplants of hematopoietic stem cells (HSCs), and immunosurveillance thereby preserving tissue integrity and function [96–99]. Remarkably, there are many factors positively regulating phagocytosis while SIRP*α*-CD47 is the only negative regulator preventing self-phagocytosis [88].

CD47 is critical for transepithelial and transendothelial migration of neutrophils or polymorphonuclear leukocytes (PMN) facilitating diapedesis through endothelial cells while targeted CD47 deletion decreases neutrophil extravasation [100, 101]. The SIRP*α*-CD47 interaction initially recruits PMNs to tumor sites or sites of injury but later negatively regulates these cells to end the inflammatory response. However, in a postacute stage of inflammation, neutrophils experience cleavage of the cytoplasmic signaling domains of SIRP*α*, correlating with increased recruitment and neutrophil-associated damage. Truncated SIRP*α* acts like a decoy, able to bind CD47 but not signaling intracellularly therefore maintaining the inflammatory microenvironment and being a caveat for CD47 targeted therapies [102–105]. Additionally, SIRP*α* binding to CD47* in vitro* downregulates CD18 as marker of neutrophil activation thus playing a role in the inflammatory activation state of PMNs [106, 107].

The dual role of CD47 in promoting inflammation through neutrophil migration and recognition of self through blocking phagocytosis in macrophages plays a role in the development of cancer and later in tumor immune evasion. Loss of CD47 induces phagocytosis by macrophages* in vitro* and blocks tumor development and metastasis* in vivo* [108]. This receptor is strongly overexpressed in several cancer types including both hematological and solid tumors [80, 91, 109, 110]. A high CD47 expression has been a poor prognostic factor for patients with these diseases [80, 111, 112]. CD47 is also highly expressed in tumor initiating cells (TICs) or cancer stem cells (CSC) where it is a marker of more aggressive tumor cells, with higher metastatic potential, and less sensitive to engulfment by macrophages, thereby escaping from immune surveillance while increasing cell proliferation through activation of the PI3K/Akt pathway [92, 113–116]. Therefore, CD47 becomes an attractive target for therapeutic approaches with both antitumor and anti-inflammatory properties and anti-CD47 antibodies are being tested with positive results in preclinical and clinical settings [80, 111, 112, 117].

In lung cancer and in several types of cancers including breast, bladder, colon, pancreatic, and hematological cancers, blocking CD47 in tumor cells leads to increased phagocytosis by macrophages and later activation of T cells [94]. The CD47:SIRP*α* interaction is involved in the pathogenesis of lung cancer and other cancer types when tumors release cytokines promoting tumor growth and stimulating the conversion of macrophages from M1 to M2 phenotype [118]. Systemic administration of nanoparticles with anti-CD47 siRNA showed efficient inhibition of lung metastasis to about 30% of controls [94]. In patients with lung metastasis, the number of circulating tumor cells (CTC) with the phenotype EPCAM(+)CD44(+)CD47(+)MET(+) were associated with poor overall survival and increased metastasis and CD47 was a marker associated with the fraction of metastasis-initiating cells within the pool of CTCs [119].

Antisense suppression of CD47 in squamous lung tumors prior to irradiation showed benefit obtaining a 71% tumor size reduction. This protection could possibly be exerted through thrombospondin-1 signaling to recover from radiation stress, revealing a strategy to protect normal tissues from radiation damage using anti-CD47 antibodies which could be useful in the application of combined radiation with targeted therapies in lung cancer [120].

**Figure 3:**
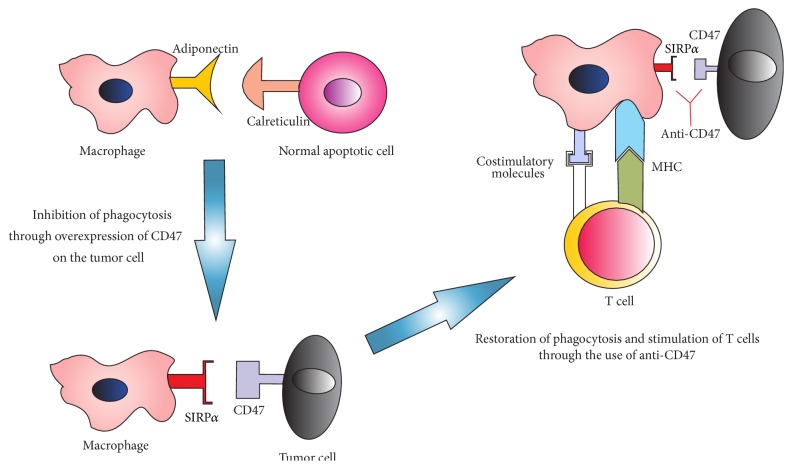
Target CD47. Macrophages maintain tissue homeostasis through phagocytosis of apoptotic cells. Interaction with other cell types can increase or inhibit their activity. Adiponectin via calreticulin leads to the uptake of early apoptotic cells by macrophages. However, as an immune evasion mechanism, tumor cells can deregulate the expression of CD47 and thereby inhibit phagocytic activity. Currently, the administration of anti-CD47 antibody activates phagocytosis by blocking interaction SIRP-*α*/CD47 and inhibits the migration of neutrophils to the tumor site inhibiting their growth.

There is a close relationship between macrophage, neutrophil infiltration, and upregulation or CD47 with poor prognosis and lack response to treatment. Nowadays, therapies are developed to block the interaction of tumor cells with macrophages through CD47, thereby offering an opportunity to turn TAMs against NSCLC cells by allowing the phagocytic behavior of resident macrophages. Also, anti-CD47 could regulate the recruitment of neutrophils into tumor and diminish the chronic inflammation Figure 3.

## 6. Therapeutic Approaches: TAMs and TANs

Preclinical studies showed that peptide to M2-like TAM improves survival of tumor bearing mouse [121]. Inhibition of CSF-1 receptor, which is essential for macrophage differentiation significantly increased survival and suppressed established tumors, accompanied by decreased M2-like TAM [122]. Treatment with metformin is able to reduce the metastases* in vivo*, through blocked matrix metalloproteinase-9 and expression of MMP-2, maintaining the components of the extracellular matrix, avoiding the separation of tumor cells, inhibiting the growth and metastasis of tumors [123]. Also, metformin prevented M2-polarization of macrophages regulated AMPK*α*1 and, besides, inhibited IL-1 induced release of the proinflammatory cytokines IL-6 and IL-8 in macrophages [124, 125]. Combination of metformin with TKI inhibitor reduces pulmonary fibrosis trough decreased TGF-beta [126].

Glycodelin (gene name PAEP) is a proliferation suppressor and apoptosis inducer of T cells, monocytes, B cells, NK, and regulated pulmonary immune response in asthmatic inflammation. However, atypical expression is observed in squamous cell carcinomas and adenocarcinomas of NSCLC [127].* In vitro*, silencing by siRNA-transfection of PAEP in two NSCLC cell lines resulted in significant upregulation of immune system modulatory factors such as PDL1, CXCL5, CXCL16, MICA/B, and CD83 as well as proliferation stimulators EDN1 and HBEGF [127]. This kind of therapy provides a mechanism to overcome tumor immunosurveillance.

As mentioned above, currently the only FDA-approved immunotherapies for the treatment of NSCLC are nivolumab and pembrolizumab. These antibodies inhibit checkpoint molecules such as CTL-4 and PDL-1, improving the survival and response to treatment [128]. CTLA-4 is thought to regulate T cell proliferation early in an immune response, primarily in lymph nodes, whereas PD-1 is upregulated in current smokers and suppresses T cells [129]. These antibodies switch on immune system cells mediated by T cells, increasing their ability to recognize and destroy cancer cells [128, 130]. Monoclonal antibodies specific for tumor cell antigens, coupled with appropriate cytokines, may provide rational basis for designing trials to employ the neutrophil cytotoxic potential as adjuvant therapy in cancer patients [131].

## 7. Conclusion

Chronic inflammation seems to play a major role in the onset and development of cancer. Understanding the interaction between the cellular and molecular factors that mediate inflammation in NSCLC, including the rather unexplored components of innate immunity such as macrophages and neutrophils, can elucidate novel targets affecting key oncogenic pathways in this malignancy and allow preventing cancer cell proliferation, angiogenesis, and metastasis. Inhibiting CD47 as promoter of neutrophil extravasation and migration may reduce inflammation thereby preventing cancer, and blocking the antiphagocytic signal of CD47 on the surface of tumor cells can overcome immune suppression, harnessing the immune system to target malignant cells more effectively. On the other hand, the potential side effects should be addressed by careful selection of patient populations based on biomarkers such as tumor CD47 overexpression.

## References

[1] Siegel R. L., Miller K. D., Jemal A. (2015). Cancer statistics, 2015. *CA: A Cancer Journal for Clinicians*.

[2] Arrieta O., Guzmán-de Alba E., Alba-López L. F. (2013). National consensus of diagnosis and treatment of non-small cell lung cancer. *Revista de Investigacion Clinica*.

[3] Jemal A., Bray F., Center M. M., Ferlay J., Ward E., Forman D. (2011). Global cancer statistics. *CA: Cancer Journal for Clinicians*.

[4] Herbst R. S., Heymach J. V., Lippman S. M. (2008). Lung cancer. *The New England Journal of Medicine*.

[5] Travis W. D. (2011). Pathology of lung cancer. *Clinics in Chest Medicine*.

[6] Devesa S. S., Bray F., Vizcaino A. P., Parkin D. M. (2005). International lung cancer trends by histologic type: male:female differences diminishing and adenocarcinoma rates rising. *International Journal of Cancer*.

[7] Boyle P., Levin B. (2008). *World Cancer Report 2008*.

[8] Sun S., Schiller J. H., Gazdar A. F. (2007). Lung cancer in never smokers—a different disease. *Nature Reviews Cancer*.

[9] Arrieta O., Rios Trejo M. A., Michel R. M. (2009). Wood-smoke exposure as a response and survival predictor in erlotinib-treated nonsmall cell lung cancer patients. *Journal of Thoracic Oncology*.

[10] Arrieta O., Ramirez-Tirado L. A., Baez-Saldana R., Pena-Curiel O., Soca-Chafre G., Macedo-Perez E. O. (2015). Different mutation profiles and clinical characteristics among Hispanic patients with non-small cell lung cancer could explain the ‘Hispanic paradox’. *Lung Cancer*.

[11] Arrieta O., Campos-Parra A. D., Zuloaga C. (2012). Clinical and pathological characteristics, outcome and mutational profiles regarding non-small-cell lung cancer related to wood-smoke exposure. *Journal of Thoracic Oncology*.

[12] Nur U., Quaresma M., De Stavola B., Peake M., Rachet B. (2015). Inequalities in non-small cell lung cancer treatment and mortality. *Journal of Epidemiology and Community Health*.

[13] Mok T. S., Wu Y.-L., Thongprasert S. (2009). Gefitinib or carboplatin-paclitaxel in pulmonary adenocarcinoma. *The New England Journal of Medicine*.

[14] Arrieta O., Anaya P., Morales-Oyarvide V., Ramírez-Tirado, L. A., Polanco A. C. (2015). Cost-effectiveness analysis of EGFR mutation testing in patients with non-small cell lung cancer (NSCLC) with gefitinib or carboplatin-paclitaxel. *The European Journal of Health Economics*.

[15] Rosell R., Carcereny E., Gervais R. (2012). Erlotinib versus standard chemotherapy as first-line treatment for European patients with advanced EGFR mutation-positive non-small-cell lung cancer (EURTAC): a multicentre, open-label, randomised phase 3 trial. *The Lancet Oncology*.

[16] Arrieta O., Martinez-Barrera L., Treviño S. (2008). Wood-smoke exposure as a response and survival predictor in erlotinib-treated non-small cell lung cancer patients: an open label phase II study. *Journal of Thoracic Oncology*.

[17] Suzawa K., Toyooka S., Sakaguchi M. (2015). Antitumor effect of afatinib, as a human epidermal growth factor receptor 2-targeted therapy, in lung cancers harboring *HER2* oncogene alterations. *Cancer Science*.

[18] Arrieta O., De la Torre-Vallejo M., Lopez-Macias D. (2015). Nutritional status, body surface, and low lean body mass/body mass index are related to dose reduction and severe gastrointestinal toxicity induced by afatinib in patients with non-small cell lung cancer. *The Oncologist*.

[19] Arrieta O., Vega-González M. T., López-Macías D. (2015). Randomized, open-label trial evaluating the preventive effect of tetracycline on afatinib induced-skin toxicities in non-small cell lung cancer patients. *Lung Cancer*.

[20] Dudnik E., Siegal T., Zach L. (2015). Durable brain response with pulse-dose crizotinib and ceritinib in ALK-positive non-small cell lung cancer compared with brain radiotherapy. *Journal of Clinical Neuroscience*.

[21] Ou S. I., Ahn J. S., De Petris L. (2015). Alectinib in crizotinib-refractory *ALK*-rearranged non-small-cell lung cancer: a phase II global study. *Journal of Clinical Oncology*.

[22] Leprieur E. G., Fallet V., Wislez M. (2015). Modalities of use of ceritinib (Zykadia^™^), a 2nd generation ALK inhibitor, in advanced stage non-small cell lung cancer. *Bulletin du Cancer*.

[23] Rosell R., Moran T., Queralt C. (2009). Screening for epidermal growth factor receptor mutations in lung cancer. *The New England Journal of Medicine*.

[24] Shigematsu H., Lin L., Takahashi T. (2005). Clinical and biological features associated with epidermal growth factor receptor gene mutations in lung cancers. *Journal of the National Cancer Institute*.

[25] Paez J. G., Jänne P. A., Lee J. C. (2004). EGFR mutations in lung, cancer: correlation with clinical response to gefitinib therapy. *Science*.

[26] Villarreal-Garza C., de la Mata D., Zavala D. G., MacEdo-Perez E. O., Arrieta O. (2013). Aggressive treatment of primary tumor in patients with non-small-cell lung cancer and exclusively brain metastases. *Clinical Lung Cancer*.

[27] Yun J. K., Kim M. A., Choi C. M. (2015). Surgical outcomes after pulmonary resection for non-small cell lung cancer with localized pleural seeding first detected during surgery. *The Thoracic and Cardiovascular Surgeon*.

[28] Liu W., Shao Y., Guan B. (2015). Extracapsular extension is a powerful prognostic factor in stage IIA-IIIA non-small cell lung cancer patients with completely resection. *International Journal of Clinical and Experimental Pathology*.

[29] La-Beck N. M., Jean G. W., Huynh C., Alzghari S. K., Lowe D. B. (2015). Immune checkpoint inhibitors: new insights and current place in cancer therapy. *Pharmacotherapy*.

[30] Soria J. C., Marabelle A., Brahmer J. R., Gettinger S. (2015). Immune checkpoint modulation for non-small cell lung cancer. *Clinical Cancer Research*.

[31] Scarpace S. L. (2015). Metastatic squamous cell non-small-cell lung cancer (NSCLC): disrupting the drug treatment paradigm with immunotherapies. *Drugs in Context*.

[32] Dang T. O., Ogunniyi A., Barbee M. S., Drilon A. (2015). Pembrolizumab for the treatment of PD-L1 positive advanced or metastatic non-small cell lung cancer. *Expert Review of Anticancer Therapy*.

[33] Patnaik A., Kang S. P., Rasco D. (2015). Phase I study of pembrolizumab (MK-3475; anti-PD-1 monoclonal antibody) in patients with advanced solid tumors. *Clinical Cancer Research*.

[34] Gunturi A., McDermott D. F. (2015). Nivolumab for the treatment of cancer. *Expert Opinion on Investigational Drugs*.

[35] Borghaei H., Paz-Ares L., Horn L. (2015). Nivolumab versus docetaxel in advanced nonsquamous non-small-cell lung cancer. *The New England Journal of Medicine*.

[36] Villadolid J., Amin A. (2015). Immune checkpoint inhibitors in clinical practice: update on management of immune-related toxicities. *Translational Lung Cancer Research*.

[37] Rossi E., Sgambato A., Chiara G. (2015). Endocrinopathies induced by immune-checkpoint inhibitors in advanced non-small cell lung cancer. *Expert Review of Clinical Pharmacology*.

[38] Lu J., Lee-Gabel L., Nadeau M. C., Ferencz T. M., Soefje S. A. (2015). Clinical evaluation of compounds targeting PD-1/PD-L1 pathway for cancer immunotherapy. *Journal of Oncology Pharmacy Practice*.

[39] Wu Y., Zhou B. P. (2009). Inflammation: a driving force speeds cancer metastasis. *Cell Cycle*.

[40] Sethi G., Shanmugam M. K., Ramachandran L., Kumar A. P., Tergaonkar V. (2012). Multifaceted link between cancer and inflammation. *Bioscience Reports*.

[41] Jaillon S., Galdiero M. R., Del Prete D., Cassatella M. A., Garlanda C., Mantovani A. (2013). Neutrophils in innate and adaptive immunity. *Seminars in Immunopathology*.

[42] Barrera L., Montes-Servín E., Barrera A. (2015). Cytokine profile determined by data-mining analysis set into clusters of non-small-cell lung cancer patients according to prognosis. *Annals of Oncology*.

[43] Carus A., Gurney H., Gebski V. (2013). Impact of baseline and nadir neutrophil index in non-small cell lung cancer and ovarian cancer patients: assessment of chemotherapy for resolution of unfavourable neutrophilia. *Journal of Translational Medicine*.

[44] Sánchez-Lara K., Turcott J. G., Juárez E. (2012). Association of nutrition parameters including bioelectrical impedance and systemic inflammatory response with quality of life and prognosis in patients with advanced non-small-cell lung cancer: a prospective study. *Nutrition and Cancer*.

[45] Oldenborg P.-A., Gresham H. D., Lindberg F. P. (2001). CD47-signal regulatory protein *α* (SIRP*α*) regulates Fc*γ* and complement receptor-mediated phagocytosis. *The Journal of Experimental Medicine*.

[46] Bingle L., Brown N. J., Lewis C. E. (2002). The role of tumour-associated macrophages in tumour progression: implications for new anticancer therapies. *The Journal of Pathology*.

[47] Mantovani A., Sica A., Locati M. (2007). New vistas on macrophage differentiation and activation. *European Journal of Immunology*.

[48] Mantovani A., Sozzani S., Locati M., Allavena P., Sica A. (2002). Macrophage polarization: tumor-associated macrophages as a paradigm for polarized M2 mononuclear phagocytes. *Trends in Immunology*.

[49] Gordon S. (2003). Alternative activation of macrophages. *Nature Reviews Immunology*.

[50] Quatromoni J. G., Eruslanov E. (2012). Tumor-associated macrophages: function, phenotype, and link to prognosis in human lung cancer. *American Journal of Translational Research*.

[51] Chang C.-I., Liao J. C., Kuo L. (2001). Macrophage arginase promotes tumor cell growth and suppresses nitric oxide-mediated tumor cytotoxicity. *Cancer Research*.

[52] Biswas S. K., Sica A., Lewis C. E. (2008). Plasticity of macrophage function during tumor progression: regulation by distinct molecular mechanisms. *The Journal of Immunology*.

[53] Yuan A., Hsiao Y. J., Chen H. Y. (2015). Opposite effects of M1 and M2 macrophage subtypes on lung cancer progression. *Scientific Reports*.

[54] Al-Sarireh B., Eremin O. (2000). Tumour-Associated Macrophages (TAMS): disordered function, immune suppression and progressive tumour growth. *Journal of the Royal College of Surgeons of Edinburgh*.

[55] Erroi A., Sironi M., Chiaffarino F., Zhen-Guo C., Mengozzi M., Mantovani A. (1989). IL-1 and IL-6 release by tumor-associated macrophages from human ovarian carcinoma. *International Journal of Cancer*.

[56] Ghezzi P., Erroi A., Acero R., Salmona M., Mantovani A. (1987). Defective production of reactive oxygen intermediates by tumor-associated macrophages exposed to phorbol ester. *Journal of Leukocyte Biology*.

[57] Lewis C. E., Leek R., Harris A., McGee J. O. (1995). Cytokine regulation of angiogenesis in breast cancer: the role of tumor-associated macrophages. *Journal of Leukocyte Biology*.

[58] Brain J. D. (1980). Macrophage damage in relation to the pathogenesis of lung diseases. *Environmental Health Perspectives*.

[59] Wang W., Liu H., Dai X. (2015). p53/PUMA expression in human pulmonary fibroblasts mediates cell activation and migration in silicosis. *Scientific Reports*.

[60] Hodge S., Hodge G., Ahern J., Jersmann H., Holmes M., Reynolds P. N. (2007). Smoking alters alveolar macrophage recognition and phagocytic ability: implications in chronic obstructive pulmonary disease. *American Journal of Respiratory Cell and Molecular Biology*.

[61] Richens T. R., Linderman D. J., Horstmann S. A. (2009). Cigarette smoke impairs clearance of apoptotic cells through oxidant-dependent activation of RhoA. *American Journal of Respiratory and Critical Care Medicine*.

[62] Subramaniam R., Mukherjee S., Chen H., Keshava S., Neuenschwander P., Shams H. (2015). Restoring cigarette smoke-induced impairment of efferocytosis in alveolar macrophages. *Mucosal Immunology*.

[63] Sone S. (1986). Role of alveolar macrophages in pulmonary neoplasias. *Biochimica et Biophysica Acta*.

[64] Bonta I. L., Ben-Efraim S. (1993). Involvement of inflammatory mediators in macrophage antitumor activity. *Journal of Leukocyte Biology*.

[65] Ohri C. M., Shikotra A., Green R. H., Waller D. A., Bradding P. (2009). Macrophages within NSCLC tumour islets are predominantly of a cytotoxic M1 phenotype associated with extended survival. *European Respiratory Journal*.

[66] Ma J., Liu L., Che G., Yu N., Dai F., You Z. (2010). The M1 form of tumor-associated macrophages in non-small cell lung cancer is positively associated with survival time. *BMC Cancer*.

[67] Lopez-Gonzalez J. S., Avila-Moreno F., Prado-Garcia H., Aguilar-Cazares D., Mandoki J. J., Meneses-Flores M. (2007). Lung carcinomas decrease the number of monocytes/macrophages (CD14+ cells) that produce TNF-*α*. *Clinical Immunology*.

[68] Mantovani A., Sica A., Locati M. (2005). Macrophage polarization comes of age. *Immunity*.

[69] Fu X., Shi H., Qi Y., Zhang W., Dong P. (2015). M2 polarized macrophages induced by CSE promote proliferation, migration, and invasion of alveolar basal epithelial cells. *International Immunopharmacology*.

[70] Kobayashi Y. (2015). Neutrophil biology: an update. *EXCLI Journal*.

[71] Seok J., Warren H. S., Cuenca A. G. (2013). Genomic responses in mouse models poorly mimic human inflammatory diseases. *Proceedings of the National Academy of Sciences of the United States of America*.

[72] Eruslanov E. B., Bhojnagarwala P. S., Quatromoni J. G. (2014). Tumor-associated neutrophils stimulate T cell responses in early-stage human lung cancer. *The Journal of Clinical Investigation*.

[73] Fridlender Z. G., Sun J., Kim S. (2009). Polarization of tumor-associated neutrophil phenotype by TGF-*β*: ‘N1’ versus ‘N2’ TAN. *Cancer Cell*.

[74] Serhan C. N., Savill J. (2005). Resolution of inflammation: the beginning programs the end. *Nature Immunology*.

[75] Mora-Jensen H., Jendholm J., Fossum A., Porse B., Borregaad N., Theilgaard-Mönch K. (2011). Technical advance: immunophenotypical characterization of human neutrophil differentiation. *Journal of Leukocyte Biology*.

[76] Colombo M. P., Ferrari G., Stoppacciaro A. (1991). Granulocyte colony-stimulating factor gene transfer suppresses tumorigenicity of a murine adenocarcinoma in vivo. *The Journal of Experimental Medicine*.

[77] Kang M. H., Go S.-I., Song H.-N. (2014). The prognostic impact of the neutrophil-to-lymphocyte ratio in patients with small-cell lung cancer. *British Journal of Cancer*.

[78] Liu Y., Merlin D., Burst S. L., Pochet M., Madara J. L., Parkos C. A. (2001). The role of CD47 in neutrophil transmigration. Increased rate of migration correlates with increased cell surface expression of CD47. *The Journal of Biological Chemistry*.

[79] Sosale N. G., Spinler K. R., Alvey C., Discher D. E. (2015). Macrophage engulfment of a cell or nanoparticle is regulated by unavoidable opsonization, a species-specific ‘Marker of Self’ CD47, and target physical properties. *Current Opinion in Immunology*.

[80] Willingham S. B., Volkmer J.-P., Gentles A. J. (2012). The CD47-signal regulatory protein alpha (SIRPa) interaction is a therapeutic target for human solid tumors. *Proceedings of the National Academy of Sciences of the United States of America*.

[81] Chao M. P., Weissman I. L., Majeti R. (2012). The CD47-SIRP*α* pathway in cancer immune evasion and potential therapeutic implications. *Current Opinion in Immunology*.

[82] Tsai R. K., Discher D. E. (2008). Inhibition of ‘self’ engulfment through deactivation of myosin-II at the phagocytic synapse between human cells. *The Journal of Cell Biology*.

[83] Brown E. J., Frazier W. A. (2001). Integrin-associated protein (CD47) and its ligands. *Trends in Cell Biology*.

[84] Lindberg F. P., Gresham H. D., Schwarz E., Brown E. J. (1993). Molecular cloning of integrin-associated protein: an immunoglobulin family member with multiple membrane-spanning domains implicated in alpha v beta 3-dependent ligand binding. *The Journal of Cell Biology*.

[85] Lindberg F. P., Bullard D. C., Caver T. E., Gresham H. D., Beaudet A. L., Brown E. J. (1996). Decreased resistance to bacterial infection and granulocyte defects in IAP-deficient mice. *Science*.

[86] Soto-Pantoja D. R., Kaur S., Roberts D. D. (2015). CD47 signaling pathways controlling cellular differentiation and responses to stress. *Critical Reviews in Biochemistry and Molecular Biology*.

[87] Barclay A. N., Van den Berg T. K. (2014). The interaction between signal regulatory protein alpha (SIRPalpha) and CD47: structure, function, and therapeutic target. *Annual Review of Immunology*.

[88] Jaiswal S., Chao M. P., Majeti R., Weissman I. L. (2010). Macrophages as mediators of tumor immunosurveillance. *Trends in Immunology*.

[89] Oldenborg P.-A., Zheleznyak A., Fang Y.-F., Lagenaur C. F., Gresham H. D., Lindberg F. P. (2000). Role of CD47 as a marker of self on red blood cells. *Science*.

[90] Jaiswal S., Jamieson C. H. M., Pang W. W. (2009). CD47 is upregulated on circulating hematopoietic stem cells and leukemia cells to avoid phagocytosis. *Cell*.

[91] Roberts D. D., Kaur S., Soto-Pantoja D. R. (2015). Therapeutic targeting of the thrombospondin-1 receptor CD47 to treat liver cancer. *Journal of Cell Communication and Signaling*.

[92] Sick E., Boukhari A., Deramaudt T. (2011). Activation of CD47 receptors causes proliferation of human astrocytoma but not normal astrocytes via an Akt-dependent pathway. *Glia*.

[93] Lawrence D. W., King S. B., Frazier W. A., Koenig J. M. (2009). Decreased CD47 expression during spontaneous apoptosis targets neutrophils for phagocytosis by monocyte-derived macrophages. *Early Human Development*.

[94] Wang Y., Xu Z., Guo S. (2013). Intravenous delivery of siRNA targeting CD47 effectively inhibits melanoma tumor growth and lung metastasis. *Molecular Therapy*.

[95] Murata Y., Kotani T., Ohnishi H., Matozaki T. (2014). The CD47-SIRP*α* signalling system: its physiological roles and therapeutic application. *Journal of Biochemistry*.

[96] Su G. H., Zhao Y. L. (2013). Role of CD47 in hematologic malignancies. *Journal of experimental hematology/Chinese Association of Pathophysiology*.

[97] Khandelwal S., Van Rooijen N., Saxena R. K. (2007). Reduced expression of CD47 during murine red blood cell (RBC) senescence and its role in RBC clearance from the circulation. *Transfusion*.

[98] Savill J., Fadok V. (2000). Corpse clearance defines the meaning of cell death. *Nature*.

[99] Greenberg S., Grinstein S. (2002). Phagocytosis and innate immunity. *Current Opinion in Immunology*.

[100] Myers S. A., DeVries W. H., Andres K. R. (2011). CD47 knockout mice exhibit improved recovery from spinal cord injury. *Neurobiology of Disease*.

[101] Liu Y., O'Connor M. B., Mandell K. J. (2004). Peptide-mediated inhibition of neutrophil transmigration by blocking CD47 interactions with signal regulatory protein *α*. *The Journal of Immunology*.

[102] Zen K., Guo Y., Bian Z. (2013). Inflammation-induced proteolytic processing of the SIRPalpha cytoplasmic ITIM in neutrophils propagates a proinflammatory state. *Nature Communications*.

[103] Lee W. Y., Weber D. A., Laur O. (2010). The role of cis dimerization of signal regulatory protein *α* (SIRP*α*) in binding to CD47. *The Journal of Biological Chemistry*.

[104] Liu Y., Soto I., Tong Q. (2005). SIRP*β*1 is expressed as a disulfide-linked homodimer in leukocytes and positively regulates neutrophil transepithelial migration. *The Journal of Biological Chemistry*.

[105] Liu Y., Bühring H.-J., Zen K. (2002). Signal regulatory protein (SIRP*α*), a cellular ligand for CD47, regulates neutrophil transmigration. *The Journal of Biological Chemistry*.

[106] Finley M. J., Rauova L., Alferiev I. S., Weisel J. W., Levy R. J., Stachelek S. J. (2012). Diminished adhesion and activation of platelets and neutrophils with CD47 functionalized blood contacting surfaces. *Biomaterials*.

[107] Stachelek S. J., Finley M. J., Alferiev I. S. (2011). The effect of CD47 modified polymer surfaces on inflammatory cell attachment and activation. *Biomaterials*.

[108] Zhao X. W., Van Beek E. M., Schornagel K. (2011). CD47-signal regulatory protein-*α* (SIRP*α*) interactions form a barrier for antibody-mediated tumor cell destruction. *Proceedings of the National Academy of Sciences of the United States of America*.

[109] Hoelzer D., Gökbuget N. (2009). T-cell lymphoblastic lymphoma and T-cell acute lymphoblastic leukemia: a separate entity?. *Clinical Lymphoma & Myeloma*.

[110] Chan K. S., Espinosa I., Chao M. (2009). Identification, molecular characterization, clinical prognosis, and therapeutic targeting of human bladder tumor-initiating cells. *Proceedings of the National Academy of Sciences of the United States of America*.

[111] Majeti R., Chao M. P., Alizadeh A. A. (2009). CD47 is an adverse prognostic factor and therapeutic antibody target on human acute myeloid leukemia stem cells. *Cell*.

[112] Chao M. P., Alizadeh A. A., Tang C. (2010). Anti-CD47 antibody synergizes with rituximab to promote phagocytosis and eradicate non-Hodgkin lymphoma. *Cell*.

[113] Naujokat C. (2014). Monoclonal antibodies against human cancer stem cells. *Immunotherapy*.

[114] Gires O. (2011). Lessons from common markers of tumor-initiating cells in solid cancers. *Cellular and Molecular Life Sciences*.

[115] Chan K. S., Volkmer J.-P., Weissman I. (2010). Cancer stem cells in bladder cancer: a revisited and evolving concept. *Current Opinion in Urology*.

[116] Ju H.-S., Li X.-J., Zhao B.-L., Hou J.-W., Han Z.-W., Xin W.-J. (1990). Scavenging effects of sodium ferulate and 18 beta-glycyrrhetic acid on oxygen free radicals. *Acta Pharmacologica Sinica*.

[117] Weissman I. (2015). Evolution of normal and neoplastic tissue stem cells: progress after Robert Hooke. *Philosophical Transactions of the Royal Society B: Biological Sciences*.

[118] Gautam P. K., Acharya A. (2014). Suppressed expression of homotypic multinucleation, extracellular domains of CD172alpha (SIRP-alpha) and CD47 (IAP) receptors in TAMs upregulated by Hsp70-peptide complex in Dalton's lymphoma. *Scandinavian Journal of Immunology*.

[119] Baccelli I., Schneeweiss A., Riethdorf S. (2013). Identification of a population of blood circulating tumor cells from breast cancer patients that initiates metastasis in a xenograft assay. *Nature Biotechnology*.

[120] Maxhimer J. B., Soto-Pantoja D. R., Ridnour L. A. (2009). Radioprotection in normal tissue and delayed tumor growth by blockade of CD47 signaling. *Science Translational Medicine*.

[121] Cieslewicz M., Tang J., Yu J. L. (2013). Targeted delivery of proapoptotic peptides to tumor-associated macrophages improves survival. *Proceedings of the National Academy of Sciences of the United States of America*.

[122] Pyonteck S. M., Akkari L., Schuhmacher A. J. (2013). CSF-1R inhibition alters macrophage polarization and blocks glioma progression. *Nature Medicine*.

[123] Chen Y.-Q., Chen G. (2015). Combined therapeutic effect and molecular mechanisms of metformin and cisplatin in human lung cancer xenografts in nude mice. *Journal of Cancer Research and Therapeutics*.

[124] Ding L., Liang G., Yao Z. (2015). Metformin prevents cancer metastasis by inhibiting M2-like polarization of tumor associated macrophages. *Oncotarget*.

[125] Isoda K., Young J. L., Zirlik A. (2006). Metformin inhibits proinflammatory responses and nuclear factor-kappaB in human vascular wall cells. *Arteriosclerosis, Thrombosis, and Vascular Biology*.

[126] Li L., Huang W., Li K. (2015). Metformin attenuates gefitinib-induced exacerbation of pulmonary fibrosis by inhibition of TGF-*β* signaling pathway. *Oncotarget*.

[127] Kunert-Keil C., Steinmüller F., Jeschke U., Gredes T., Gedrange T. (2011). Immunolocalization of glycodelin in human adenocarcinoma of the lung, squamous cell carcinoma of the lung and lung metastases of colonic adenocarcinoma. *Acta Histochemica*.

[128] Buchbinder E. I., Desai A. (2015). CTLA-4 and PD-1 pathways: similarities, differences, and implications of their inhibition. *American Journal of Clinical Oncology*.

[129] D'Incecco A., Andreozzi M., Ludovini V. (2015). PD-1 and PD-L1 expression in molecularly selected non-small-cell lung cancer patients. *British Journal of Cancer*.

[130] Beckers R. K., Selinger C. I., Vilain R. (2015). PDL1 expression in triple-negative breast cancer is associated with tumour-infiltrating lymphocytes and improved outcome. *Histopathology*.

[131] Dallegri F., Ottonello L. (1992). Neutrophil—mediated cytotoxicity against tumour cells: state of art. *Archivum Immunologiae et Therapiae Experimentalis*.

